# Effects of tree species identity on soil microbial communities in *Juglans nigra* and *Quercus rubra* plantations

**DOI:** 10.3389/fmicb.2024.1442026

**Published:** 2024-10-29

**Authors:** Juan P. Frene, Shaneka S. Lawson, Niall D. Lue Sue, Ralph H. Crawford, Terrence G. Gardner

**Affiliations:** ^1^Department of Crop and Soil Sciences, North Carolina State University, Raleigh, NC, United States; ^2^USDA Forest Service, Northern Research Station, Hardwood Tree Improvement and Regeneration Center (HTIRC), Department of Forestry and Natural Resources, Purdue University, West Lafayette, IN, United States; ^3^Northern Research Station, USDA Forest Service, Wyndmoor, PA, United States

**Keywords:** forestry, microbiome, plants, soil, soil ecology

## Abstract

Understanding how different tree species affect soil microbial communities is crucial for sustainable forestry and ecosystem management practices. Despite Black walnut (*Juglans nigra* L.) forestry having a rich history, the overall comprehension of how this hardwood species influences soil remains incomplete. In earlier studies, we examined the effects of hardwood plantations on soil chemical properties and their interaction with microbial biomass, however, we highlight the importance of studying the soil microbial communities and their relationship with soil properties in greater depth. Building on this foundation, our research focused on evaluating microbiome compositions beneath *J. nigra* and another hardwood, Northern red oak (*Quercus rubra* L.) after a decade of establishment. We uncovered intriguing patterns within the soil bacterial/archaeal and fungal structures by conducting meticulous analyses utilizing amplicon sequencing alongside soil chemical properties. Our findings underscore that tree species play a pivotal role in shaping soil microbial structures, a role that surpasses even seasonal and depth influences. Most notably, *J. nigra* stands out for its ability to enhance microbial diversity, as evidenced by increased alpha-diversity indices compared to baseline values. Conversely, *Q. rubra* tends to decrease these indices. Significant disparities in microbial composition between the two tree species were evident, with *J. nigra* exhibiting enrichment in certain taxa such as *Nitrospira*, *Geobacter*, and *Bacillus* while *Q. rubra* showed enrichment in others like *Acidobacteriota* and ectomycorrhizal fungi. Furthermore, we also observed differences in co-occurrence networks by delving deeper into the interconnections within the soil microbiota. In both fungal and bacterial/archaeal communities, *J. nigra* and *Q. rubra* notably decreased the number of connections within their networks, while *Q. rubra* increased some, suggesting a more interconnected network. These differences were further highlighted by network metrics with *Q. rubra* displaying a higher mean degree and clustering coefficient. Additionally, our analysis revealed that tree species influence soil chemical properties, either directly or indirectly, thereby affecting soil bacterial and fungal communities. In conclusion, our study elucidates the intricate interplay between tree species and soil microbiota, emphasizing the need to consider these relationships in forestry and ecosystem management practices.

## 1 Introduction

The soil on the planet Earth teems with life and harbors a remarkable diversity of microorganisms which constitute over 15% of Earth’s biomass (Wall et al., 2019). These microorganisms, collectively known as microbiota, are involved in vital ecological processes such as biogeochemical cycles, carbon (C) mineralization, and plant nutrient acquisition (Fierer, 2017). These complex relationships are intricately intertwined with the diverse biological components of soil communities (Tecon and Or, 2017). Various environmental factors like pH, substrate quality, temperature, and water availability sculpt the intricacies of microbial interactions. Moreover, the dynamic nature of microbial community composition spans from short-term seasonal fluctuations to long-term changes over years or decadal scales, adding another layer of complexity to the soil ecosystem (Morriën et al., 2017). The depth of soil further influences microbial communities, revealing gradients in soil resource availability and pH that correlate with variations in the microbiome (Tang et al., 2018).

Bacteria, archaea, and fungi form taxonomically and functionally diverse communities within the soil and rhizosphere that facilitate plant nutrient uptake through direct access to limiting nutrients (Ling et al., 2022). These communities are interconnected through intricate networks, which comprise interactions within and across species and domains (Wassermann et al., 2019). Furthermore, microorganisms also influence ecosystem dynamics by mediating the exchange of compounds between plant vegetation and soils. The interactions between trees and microorganisms within forest ecosystems are vital to shaping soil microbial communities as these relationships influence ecosystem dynamics and nutrient cycling (Ferlian et al., 2021).

Forests, especially in the northern hemisphere, emerge as critical sinks with tree-microorganism interactions (Onufrak et al., 2020): trees harbor specific microorganisms that perform essential functions such as nutrient supply and stress protection, thereby shaping plant health and tree species distribution (Bakker et al., 2020; Pantigoso et al., 2022; Sánchez-Cañizares et al., 2017). The forest environment significantly influences soil microbiomes through aboveground (e.g., leaf litter) and belowground (e.g., rhizodeposition) contributions (Reich et al., 2005; Calvaruso et al., 2010). Symbiotic relationships between plants and microorganisms, including those between bacteria and fungi, play a pivotal role in nutrient and water access (Ramirez et al., 2019; Averill et al., 2019). These differences underscore the necessity of understanding microbial composition at varying depths and over time to appreciate the complex interactions within forest soils fully. Forest soils are predominantly inhabited by fungal communities, with associations varying based on tree mycorrhizal type (Lauber et al., 2009; Averill et al., 2019). Each tree species exhibits distinct rhizodeposition and leaf litter patterns that selectively enhance or suppress soil microbial members (Baldrian, 2017).

Black walnut (*Juglans nigra* L.) and Northern red oak (*Quercus rubra* L.) are notable timber species that contribute distinct characteristics to soil conditions (Williams, 1990; Dyderski et al., 2020). Although both tree species influence soil microbial communities but differ in their effects on soil functions and microbial associations (Washington, 2003; Mortier et al., 2020; Gardner et al., 2023). For instance, *J. nigra* enhances microbial biomass and soil functions, whereas *Q. rubra* may have opposing effects (Sun et al., 2013; Gardner et al., 2023). Their associations with different tree mycorrhiza types (AM for *J. nigra* and EM for *Q. rubra*) also contribute to distinct soil microbial dynamics (Washington, 2003; Mortier et al., 2020; Gardner et al., 2023).

In our study, we sought to elucidate the specific effects of *J. nigra* and *Q. rubra* on soil structure. Specifically, our interests involve investigating the composition of bacterial, archaeal, and fungal communities within these plantations a decade post-establishment. We hypothesized that (i) tree species strongly shaped microbial communities in the soils of each forest plantation as a consequence of a change of soil pH; (ii) soil depth will enhance more *Proteobacteria* and *Bacteroidota* due to litter addition in the upper layer while *Acidobacteroidota* is more abundant in deeper layers as a consequence of less nutrient availability, and (iii) seasonal changes on soil temperature and humidity will affect more bacterial communities than fungal communities. To test our hypothesis, we investigated the structure of soil bacterial/archaeal (16S rRNA) and fungal (ITS) communities, using Illumina Miseq sequencing, at two different time points: autumn (October) and spring (March), and two soil depths. Additionally, we analyzed microbial structural and belowground C and N transfer, shaped by trees and through microbial processes. To unravel the intricate relationships within soil microbial communities, we employed co-occurrence networks and evaluations of soil chemical parameters to assess their responses to environmental factors such as seasonality and soil depth variations. Ultimately, our findings contribute to the broader understanding of the complex interplay between tree species and soil microbiomes.

## 2 Methods

### 2.1 Soil site

Our investigation was conducted within a *J. nigra* and *Q. rubra* forest plantation located near Calcedonia, in midwestern Michigan (42.8412°N, 85.5828°W). The plantation, established in 2008, maintained a 2 m spacing between each tree. Site selection was carefully planned to cover the growth areas of both tree species, ensuring a thorough examination of soil microbial communities. We selected plots for analysis that encompassed each planting. Four composited samples were obtained from adjacent blocks, each confined to a 20 × 20 m area. Because of the near canopy and surface litter, there was no extra vegetation in the *Q. rubra*’s planted plot. On the other hand, the *J. nigra*’s planted plot had scant vegetation made up of local grasses. Additionally, a site devoid of plants (DB) adjacent to these blocks served as a reference for detecting changes and was centrally situated between the two sites. The soil in the area is predominantly Blount loam, with a slope averaging 1–3% but ranging from 0 to 6 percent. The texture of the soils was found to be silty clay loam or clay loam till. The average annual temperature at the study site is approximately 9.5°C, with an annual precipitation of 932 mm.

Soil samples were collected in October 2019 and March 2020 using a 2.5 cm diameter soil corer. Cores were taken 1 m away from the trunk in each plot. Surface litter was removed to ensure consistent sample collection as plot surfaces were covered in fallen leaves during the autumn sampling, a condition absent during the spring. In each case, plots were spaced at least 50 m apart, with a total of 4 plots per tree species, each adhering to the standard 20 × 20 m dimensions. Our composite soil sample was created from 25 randomly selected soil cores across each plot. Samples were collected from two soil depths, 0–10 and 10–20 cm, before being promptly transported to the laboratory on freezer packs to preserve microbial integrity. Upon arrival, all soil samples were sieved through a 2-mm mesh, with small roots discarded. After this, samples were split into two, one part was stored at −20°C for DNA extraction, and the other half was air dried for soil properties characterization.

### 2.2 Characterization of soil properties

We conducted a series of laboratory analyses, to thoroughly evaluate the soil properties across all samples which included assessments of gravimetric soil moisture, total carbon (TC), total organic carbon (TOC), total nitrogen (TN), and pH (H₂O). Gravimetric soil moisture was first determined by extracting 10 g subsamples, which were subsequently dried in an oven at 105°C for 24 h. These dried samples were then utilized for soil dry weight correction. TOC, TC, and TN were quantified using automated dry combustion, performed with a LECO Tru-Spec CN analyzer at the Instrumentation and Environmental Laboratory Services (IELS) at North Carolina State University (NCSU), located in North Carolina, USA. To assess soil pH, we employed a compound electrode from (Accumet, MA, USA) following the method described by Smith and Mullins (1991) that utilized a soil-water ratio of 1:2.5.

### 2.3 DNA extraction and PCR amplification

Soil DNA extraction was conducted in triplicate using 0.3 g of soil (total fresh weight) with the FastDNA™ SPIN Kit for Soil (MP Biomedicals, Solon, OH, USA), following the manufacturer’s protocol to accurately analyze microbial communities. Subsequently, we targeted the fungal ITS1 hypervariable region for amplification via PCR, utilizing the primers ITS1F (5′-CTTGGTCATTTAGAGGA AGTAA-3′) and ITS2R (5′-GCTGCGTTCTTCATCGATGC-3′) for fungal community analysis (Yan et al., 2021). Bacterial and archaeal communities were characterized with barcoded fusion primers 341F (CCTACGGGNGGCWGCAG) and 805R (GACTACHVGGGTAT CTAATCC) to amplify and sequence the V3–V4 region of the 16S rDNA gene (Herlemann et al., 2011). The PCR protocol began with a 3 min denaturation step at 95°C, followed by 27 cycles of 30 s denaturation at 95°C, a 30 s annealing at 55°C, and a 45 s elongation at 72°C before concluding with a 10 min extension at 72°C. Reactions were performed in triplicate. Each 20 μL reaction volume contained 2 μL of 2.5 mM dNTPs, 0.8 μL each of the forward and reverse primers, 4 μL of 5× FastPfu Buffer, (5 μM), 0.4 μL of FastPfu Polymerase (TransGen Biotech), and 10 ng of template DNA.

### 2.4 Illumina MiSeq sequencing

Following agarose gel electrophoresis, amplicons were extracted and purified using the AxyPrep DNA Gel Extraction Kit (Axygen Biosciences, Union City, CA, USA). The purified amplified PCR products were then quantified with a QuantiFluor™-ST Fluorometer (Promega, USA). Next-generation sequencing was conducted at the NC State University Genomic Sciences Laboratory (Raleigh, NC, USA) utilizing Illumina MiSeq technology, employing a paired-end sequencing approach with 2 × 300 bp read lengths.

### 2.5 Bioinformatics and data analysis

Raw reads were processed using Mothur v.1.48.1 software (Schloss, 2020) for comprehensive bioinformatics and computational analysis. Trimming operations adhered to specific criteria, requiring a minimum length of 425 and 350 bp for bacterial and fungal amplicons, a minimum quality score of 25, allowance for one primer mismatch, and no barcode mismatches. Additionally, homopolymers greater than 10 were restricted. Reads with indistinct bases and singletons were eliminated, and chimeras were detected and eliminated using Uchime (Version 4.2) within the Mothur platform (Edgar, 2013). Subsequently, alignment and classification of 16S rDNA and ITS sequences were performed against the SILVA bacterial SSU reference database v138.2 (Yilmaz et al., 2014) and UNITE v10 database (Abarenkov et al., 2023), respectively. The resultant cleaned and refined sequences were grouped into distinct operational taxonomic units (OTUs) through the Average-Neighbor clustering algorithm – UPGMA implementation in Mothur, with a 97% minimum sequence similarity criterion. The average sequence count per sample was 160,813 for 16S rDNA and 135,690 for ITS sequences. Rarefaction was conducted using the Mothur program to standardize sample sizes to the smallest number of sequences (66,066 for 16S rDNA and 68,492 for ITS sequences) using the ‘sub.sample’ command (Supplementary Figure 1). Diversity Shannon index between samples was fair and accounted for differences in sequencing depth.

### 2.6 Classification of fungal functional guilds

Fungal operational taxonomic units (OTUs) were then assigned to fungal functional guilds using the FungalTraits database (Pölme et al., 2020). OTUs assigned for more than one functional guild were analyzed by dividing the sequence counts of each OTU by the number of functional guilds assigned to that OTU. This approach allowed for visualization of differences in predicted fungal function between tree species. Stacked bar plots depicting the relative abundance of each fungal functional guild were created for each tree species, according to season, and depth.

### 2.7 Statistical analysis

Data interpretation and graphical representation were obtained using the R software environment (version: 4.1.2, R Core Team, 2017), and all the figures were designed using *ggplot2* package (v3.5.0) in R (Wickham, 2016). Differences in soil nutrient content and microbial community diversity (Shannon alpha-diversity index; α-diversity) were assessed through analysis of variance (ANOVA) with Tukey’s post hoc test (*stats*, v4.3.0, and *agricolae*, v1.3–7, packages). Nonmetric dimensional scaling (NMDS) ordinations based on Bray–Curtis dissimilarity matrices aided in visualizing beta-diversity (β-diversity) patterns (*ordinate* function, *phyloseq* package, v1.46.0). Treatment effects on β-diversity were assessed utilizing permutation-based analysis of variance (PERMANOVA), with subsequent multiple comparisons and Bonferroni adjustment to determine differences in community composition (*adonis* function, *vegan* package, v2.4–6). The Shannon diversity index was calculated using normalized OTU data (*phyloseq* package). Indicator species analyses were conducted to identify bacterial/archaeal and fungal OTUs indicative of tree species, soil depth, and seasonal variations (De Cáceres, 2013). Validation and identification of taxa responsible for observed variations were performed through Generalized Linear Model (GLM) fitting using the negative binomial distribution via the DESeq function of the *DESeq2* package (v1.42.0) in R (Love et al., 2014). Visual representations of taxa abundance differences between treatments were generated using the *phyloseq* package (McMurdie and Holmes, 2013). The validity of the differential abundance analysis was confirmed by employing Analysis of Compositions of Microbiomes with Bias Correction (ANCOM-BC, v2.4.0), ensuring a robust assessment of microbial absolute abundances (Lin and Peddada, 2020). Microbial co-occurrence patterns across tree species and the reference sample (DB) were investigated using the SpiecEasi package (v1.0.0) in R (Kurtz et al., 2015), and the sparCC function (*SpiecEasi* package) was used to calculate the correlation between each genus. The genus with a frequency lower than 75% and sparCC correlation values less or equal to 0.6 were removed from the network to reduce artificial correlation. Primary keystone microbes were identified based on their betweenness centrality and degree values within the bacterial and fungal networks (Vick-Majors et al., 2014; Tipton et al., 2018). Keystone taxa in the bacterial network had degrees surpassing 100 and betweenness centrality scores exceeding 800, while in the fungal network, keystone taxa had degrees above 3 and betweenness centrality scores above 150 (Kourtellis et al., 2013). Network complexity was assessed based on average connectivity, clustering coefficient, and edge number using the *vegan* and *igraph* packages (v1.6.0) in R (Shi et al., 2016; Banerjee et al., 2019). Mantel test and Spearman correlation were used to correlate the bacterial/archaeal and fungal communities with chemical, physical, and biochemical parameters.

### 2.8 Data availability

All sequencing data (raw) for bacterial and fungal reads have been archived in the Sequence Read Archive (SRA) of the National Center for Biotechnology Information (NCBI) under BioProject number PRJNA1035935. Additionally, the count tables, taxonomic tables, and metadata tables generated during this study are accessible at https://zenodo.org/record/10070239. Furthermore, the R scripts utilized for data analysis and processing are openly available at https://github.com/JuanFrene/Black-Walnut. These resources are provided to facilitate transparency, reproducibility, and further investigation by the scientific community.

## 3 Results

### 3.1 Soil environmental parameters under different tree species, soil depth, and season

A summary of the environmental properties assessed in this research is found in Table 1. Significant variations were observed in TOC (*p* = 0.0018), TC (*p* < 0.0001), C to N ratio (C/N) (*p* = 0.0482), and TN (*p* = 0.0002), primarily influenced by tree species. In particular, *J. nigra* exhibited higher levels of these parameters compared to *Q. rubra* (Table 1). Seasonal analysis further revealed that TOC (*p* = 0.0424), TC (*p* = 0.0424), and soil humidity (*p* = 0.0088) were significantly higher during autumn and in the upper soil layer (0–10 cm). TN levels, on the other hand, were consistently greater in the upper layer regardless of season (*p* = 0.0424) (Table 1). Furthermore, dramatic differences in pH levels were observed, with *Q. rubra* exhibiting a lower pH than *J. nigra* (*p* < 0.0001) and the control site DB (Table 1).

**Table 1:** Mean values of soil chemical properties by tree species.

Tree species	Hum	pH	TOC	TC	TN	C:N
*J. nigra*	19.76**a**	6.66**a**	1.60	1.84**a**	0.16**a**	11.88
*Q. rubra*	17.57**b**	5.81**b**	1.14	1.19**b**	0.11**b**	10.74
DB	19.08**a**	6.86**a**	1.42	1.62**ab**	0.14**ab**	11.32

Mean values followed by different letters are significantly different at the *p* < 0.05 level.

BD, non-plant soil; Hum, Humidity; TOC, Total organic carbon; TC, total carbon; TN, total nitrogen; and C:N, carbon and nitrogen ratio.

### 3.2 Overall characteristics of the soil microbiome

**Figure 1:**
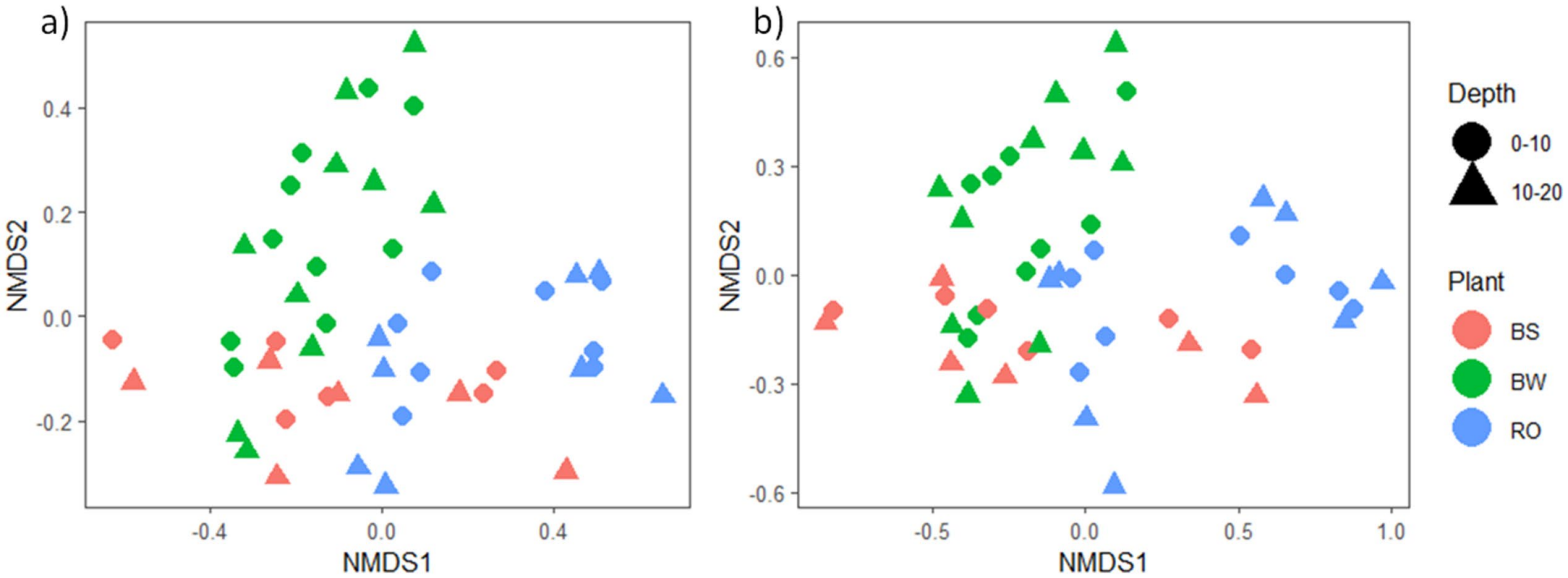
Non-metric multidimensional analysis (NMDS) plot illustrating the influence of tree species on **(a)** bacterial/archaeal and **(b)** fungal community composition. Each character corresponds to a sample collected in each season, while each color represents a category (*J. nigra*, *Q. rubra*, or DB).

**Figure 2:**
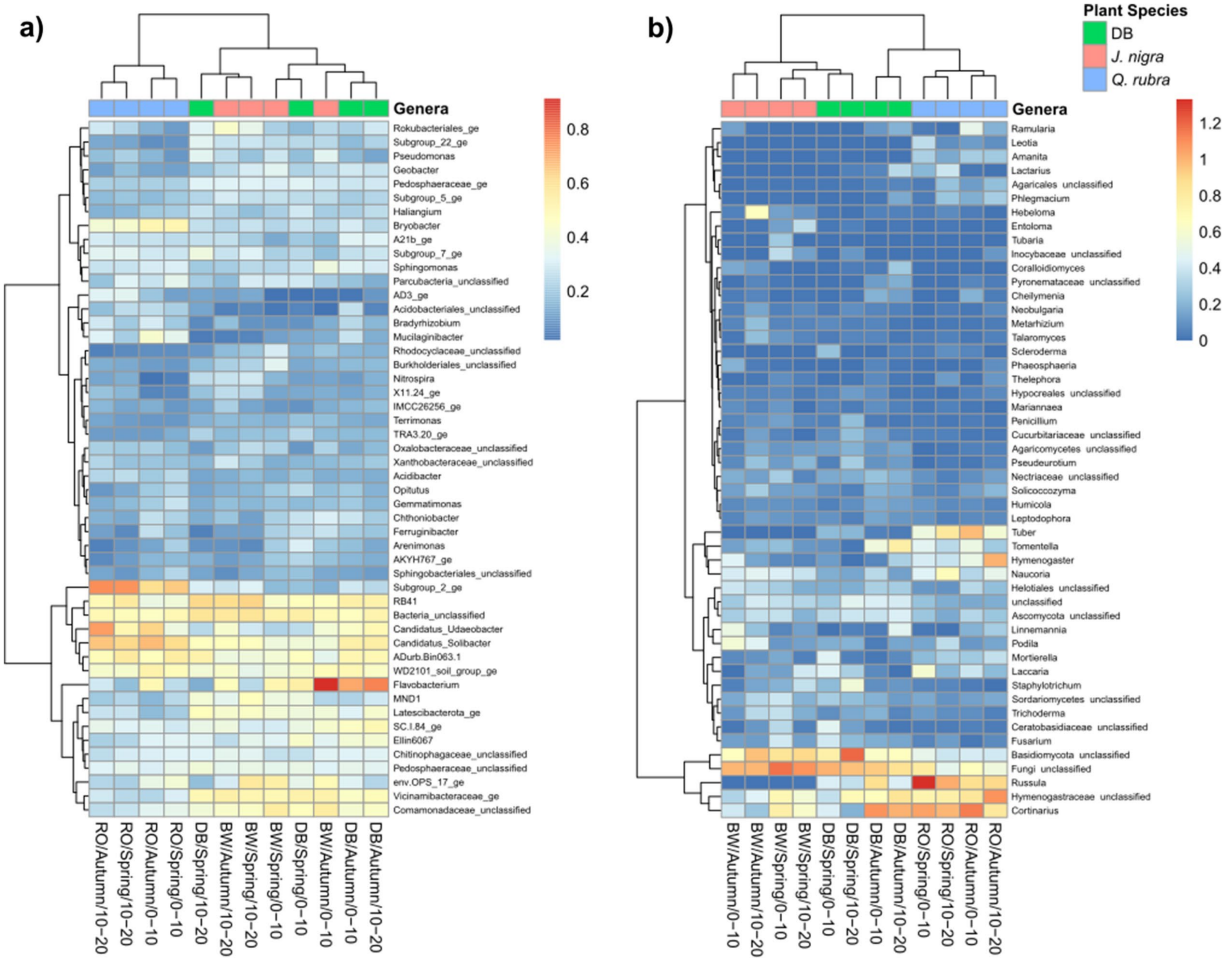
Heatmap showing the abundances of the 50 most abundant **(a)** bacterial/archaeal genera and **(b)** fungal genera in the soils for all three categories by depth, and season. Data were centered and scaled to the mean of each taxon’s log-transformed relative abundance. Samples are clustered along the top x-axis dendrogram based on community composition, while taxa are clustered on the side y-axis dendrogram according to their relative abundance distributed across samples. DB, non-plant soil; BW, *J. nigra*; RO, *Q. rubra.*

**Figure 3:**
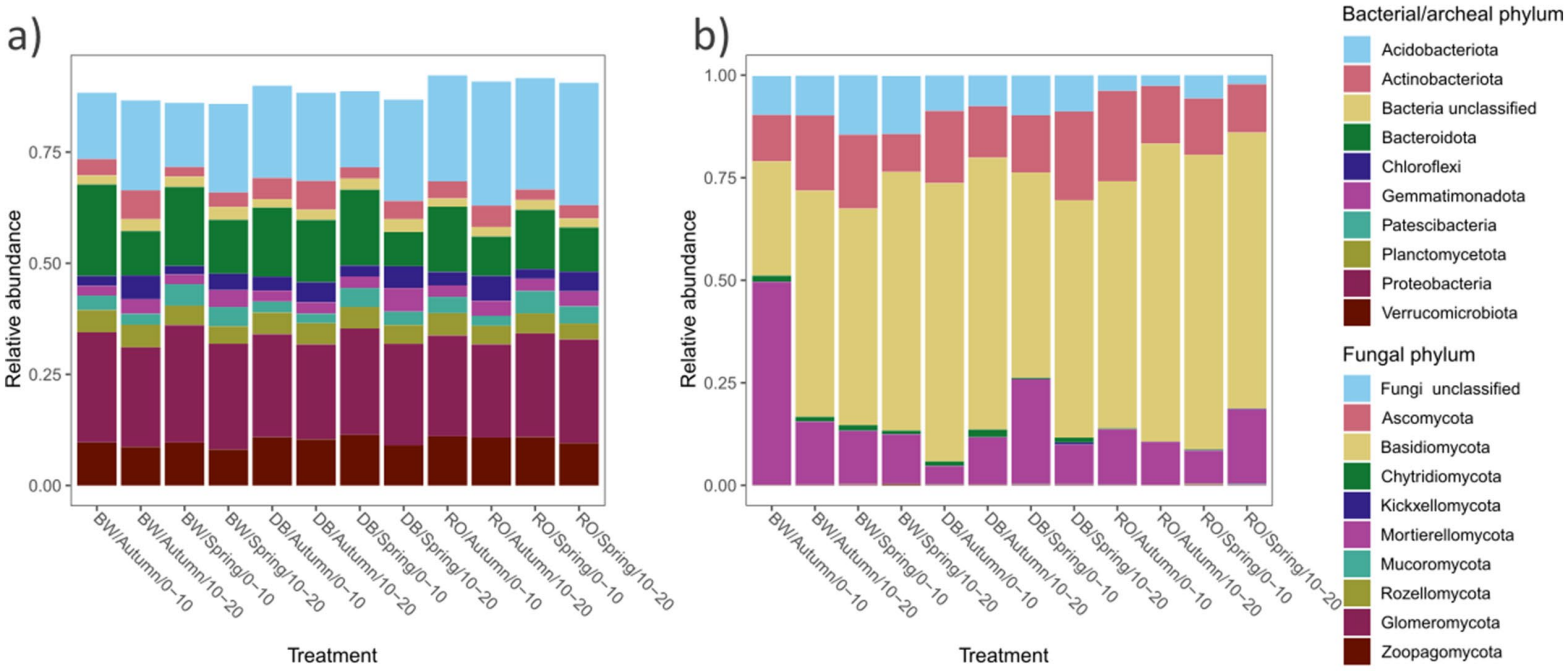
Relative abundances of **(a)** bacterial/archaeal and **(b)** fungal taxa, estimated from the proportional abundances of classifiable sequences, excluding unclassified sequences below the domain level. DB, non-plant soil; BW, *J. nigra*; RO, *Q. rubra*.

We conducted NMDS ordination based on Bray-Curtis distances and subsequently performed PERMANOVA analysis to highlight seasonal and species-specific variation in microbial community structure to elucidate the factors influencing microbial community composition. Tree species emerged as the strongest predictor of bacterial/archaeal community composition (*p* = 0.001), followed by depth (*p* = 0.002) and season (*p* = 0.015, Figure 1a). Specifically, tree species accounted for 17.8% of bacterial/archaeal community variation, while depth and season contributed 4.2 and 6.2%, respectively (Figure 1a). Notably, both *J. nigra* and *Q. rubra* shared approximately 20% of unique bacterial/archaeal OTUs, with 26 and 20% unique OTUs, respectively. In contrast, DB harbored 15% unique bacterial/archaeal OTUs (Supplementary Figure 2a). Genus-level relative abundance data illustrated that *Acidobacteriota* and *Proteobacteria* were the most abundant taxa (Figure 2a). Specifically, *Subgroup_2_ge*, *Candidatus_Solibacter* (both *Acidobacteriota*) and *Candidatus_Udaeobacter (Verrucomicrobiota)* exhibited greater relative abundance in *Q. rubra*, whereas *Flavobacterium* (*Bacteroidota*), *Vicinamibacteraceae_ge*, and *Comamonadaceae_unclassified* (*Proteobacteria*) were more prevalent in DB and *J. nigra* (Figure 2a). The most pronounced differences were observed between *Q. rubra* and the other treatments. *J. nigra* and DB samples were primarily distinguished by season while depth exerted a greater influence on *Q. rubra* samples. Moreover, bacterial/archaeal α-diversity, as indicated by the Shannon index, was significantly higher under *J. nigra* for both seasons and depths compared to *Q. rubra* and DB (Figures 3a,b). Spring values of α -diversity generally exceeded those in autumn though no significant differences were detected across depth or season and no significant interactions among these factors were noted (*p* > 0.05) (Figures 3a,b).

Tree species also significantly influenced fungal communities (*p* = 0.001), followed by soil depth (*p* = 0.001) (Figure 1b). A weak but significant interaction was observed relating to tree species and season (*p* = 0.049). Tree species accounted for 12.6% of fungal community variation, while depth and season explained 3% (Figure 1a). Moreover, *J. nigra* and *Q. rubra* shared 7% of unique fungal OTUs, with *J. nigra* and *Q. rubra* accounting for 34.75 and 22.25%, respectively. In contrast, DB contained 21.32% unique fungal OTUs (Supplementary Figure 2b). Genus-level relative abundance data exhibited variability across tree species, season, and soil depth (Figure 2b). *Q. rubra* exhibited a higher abundance of *Cortinarius*, Tuber, Russula, *Lactarius*, and *Ramularia* while *J. nigra* exhibited a higher abundance of *Linnemannia*, *Podila*, *Trichoderma*, and *Fusarium* (Figure 2b). Fungal α-diversity, as measured by the Shannon index, showed significant differences related to tree species (*p* = 0.0004), depth, and season (Figures 3c,d). Specifically, *J. nigra* exhibited the highest α-diversity, followed by *Q. rubra* and DB (Figure 3c). No significant differences were observed across depth or season, nor were there significant interactions among these factors (*p* > 0.05).

The analysis of fungal communities allowed for an exploration of functional guilds’ distribution and an assessment of their response to different environmental factors. Among fungal sequences, 1,046 of 7,893 OTUs were ascribed to a particular fungal functional guild. Ectomycorrhizas (EcM) were the most commonly identified guild, followed by saprotroph communities (Supplementary Figure 3). No significant differences in fungal guild relative abundances were observed between treatments, depth, or season (*p* > 0.05). However, significant differences in the richness of fungal guilds between tree species were noted. Saprotroph richness was notably higher in *J. nigra* (*p* = 0.0315), while *Q. rubra* exhibited greater EcM richness (*p* < 0.001). Additionally, saprotroph diversity showed significant seasonal variation (*p* = 0.0178), with greater diversity observed during autumn (Supplementary Table 1).

### 3.3 Major prokaryotic and fungal taxa

**Figure 4:**
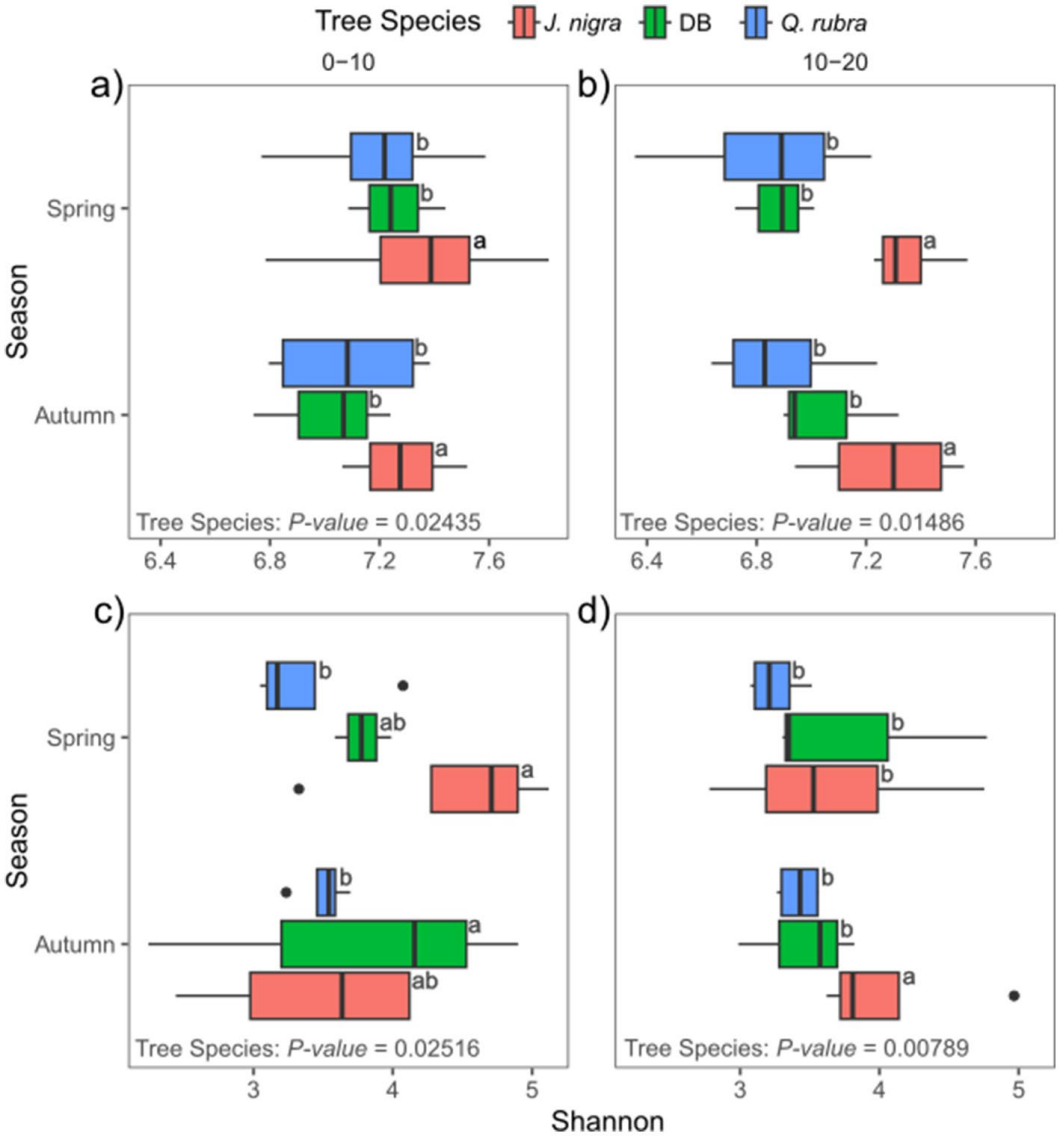
Influence of tree species, season, and depth on *α*-diversity for **(a,b)** bacteria/archaeal communities and **(c,d)** fungal communities at 0–10  cm **(a,c)** and 10-20  cm **(b,d)** soil depths. Values with different letters are significantly different among seasons (*p*-value < 0.05; ANOVA, followed by Tukey post-hoc test). DB, non-plant soil; BW, *J. nigra*; RO, *Q. rubra*.

In the *J. nigra*, *Q. rubra*, and DB samples, *Proteobacteria* emerged as the dominant phylum among the recovered bacteria/archaea sequences, comprising 23.2% of the total, followed by *Acidobacteriota* (21.2%), *Bacteroidota* (13.4%), *Verrucomicrobiota* (9.9%), *Planctomycetota* (4.5%), and *Actinobacteriota* (3.8%) (Figure 4a). These top 6 phyla collectively accounted for over 90% of the recovered sequences. A total of 54 phyla were identified in the soil samples. At the class level, *Gammaproteobacteria* and *Alphaproteobacteria*, both belonging to the *Proteobacteria* phylum, represented 15.29 and 7.97% of the recovered 16S sequences, respectively. *Proteobacteria* exhibited significant variations associated with soil depths, while *Acidobacteriota*, *Bacteroidota*, and *Myxococcota* displayed variations linked to species and soil depth (Figure 4a). Other phyla such as *Actinobacteriota* and *Planctomycetota* were significantly influenced across depth and season. Notably, *Armatimonadota* were significantly influenced by tree species, while *Chloroflexi* showed a significant correlation with soil depth (Figure 4a).

Among the recovered fungal sequences from *J. nigra*, *Q. rubra*, and DB, *Basidiomycota* emerged as the predominant phylum, accounting for 59.3% of the total, followed by *Ascomycota* (15.3%), and *Mortierellomycota* (16.2%) (Figure 4b). Together, these phyla represented over 90% of the recovered sequences. *Basidiomycota*, *Aphelidiomycota*, and *Chytridiomycota* were significantly influenced by tree species, whereas *Kickxellomycota* was predominantly influenced by seasonal variations (Figure 4b). *Mortierellomycota* exhibited variations primarily associated with tree species that were evident only in autumn (Figure 4b).

### 3.4 Indicator species analysis

**Figure 5:**
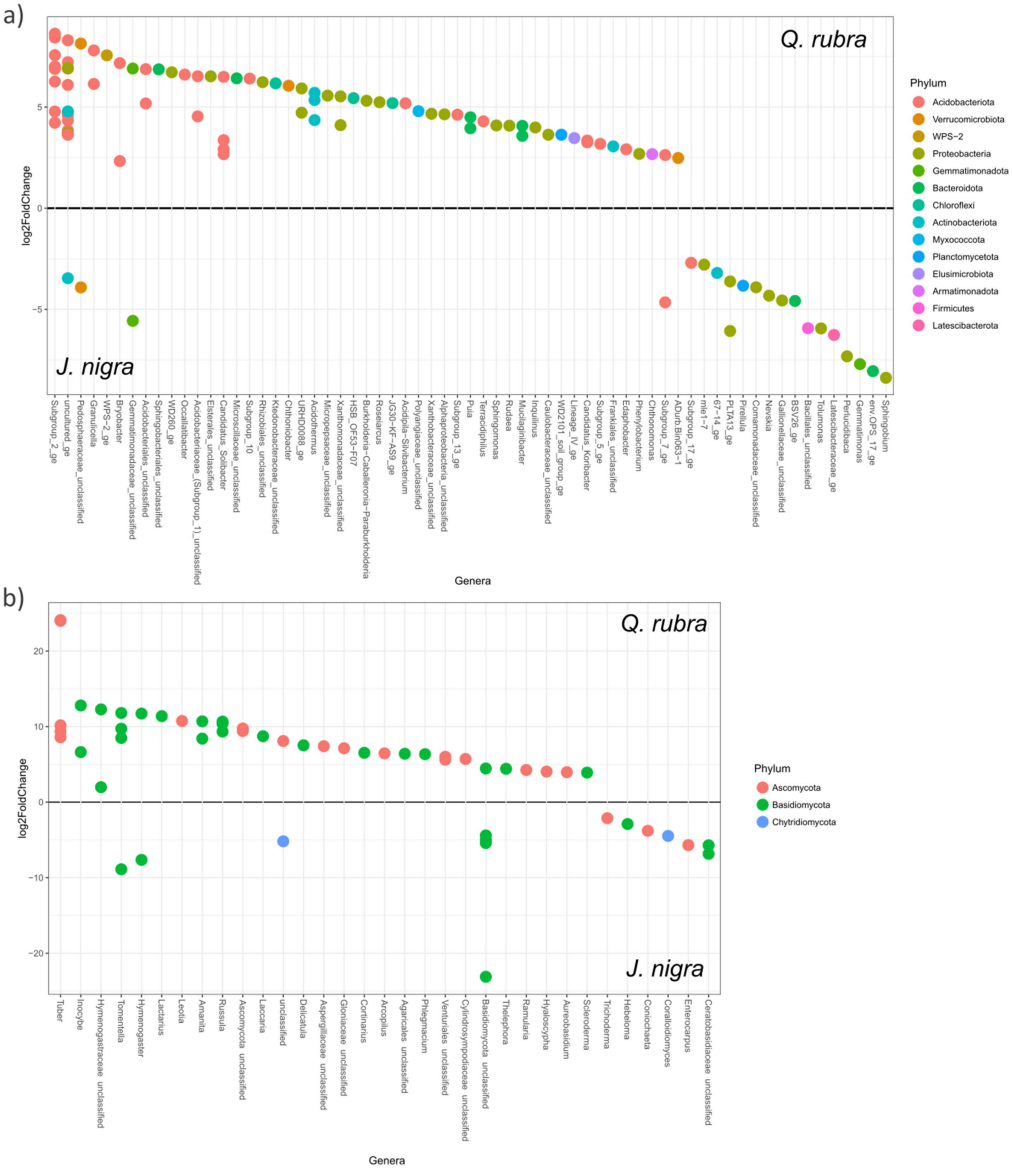
Differential representation of significant abundance OTUs, based on DESeq, between *J. nigra* and *Q. rubra* at the genus level for **(a)** bacterial/archaeal and **(b)** fungal taxa. Dots represent taxa, with colors indicating phylum and labels representing genus. Multiple points per genus correspond to different species of the genus. Positive values indicate a positive response to *Q. rubra*. Negative values indicate a positive response to *J. nigra*. Only OTUs with differential abundance at *p* < 0.05 are shown.

A total of 1,680 indicator operational taxonomic units (OTUs) were identified across the three designations: *J. nigra* (498 OTUs), *Q. rubra* (629 OTUs), and DB (256 OTUs) for bacterial/archaeal communities. Notably, *J. nigra* exhibited a higher percentage of *Bacteroidota* and *Planctomycetota*, while *Q. rubra* displayed elevated levels of *Acidobacteriota* (primarily *Acidibacter*, *Bryobacter*, and *Subgroup_2_ge*), *Armatimonadota*, and *Planctomycetota* compared to DB (Supplementary Table 2). Within *Proteobacteria*, *J. nigra* increased α- and β-*Proteobacteria*, whereas *Q. rubra* specifically elevated α-*Proteobacteria* compared to DB. Conversely, the presence of *J. nigra* led to a reduction in *Actinobacteriota* and *Chloroflexi* percentages, while *Q. rubra* limited the presence of *Firmicutes* (Supplementary Table 2). DESeq analysis corroborated these findings, highlighting significant taxonomic distinctions observed in *J. nigra* and *Q. rubra* (Figure 5a). This analysis revealed a total of 110 different OTUs belonging to 12 different phyla. The most abundant taxa within *Q. rubra* included *Subgroup_2_ge*, *Granulicella*, *Bryobacter*, *Occallatibacter*, *Candidatus_Solibacter*, and the *Pedosphaeraceae_unclassified*. Conversely, prevalent responders in *J. nigra* encompassed *Sphingobium*, *Gemmatimonas*, *Tolumonas*, *Bacillales_Unclassified*, and *Perlucidibaca* (Figure 5a). Comparing *Q. rubra* to DB unveiled pronounced differences primarily in *Acidobacteriota* and β-*Proteobacteria* taxa (Supplementary Figure 4a). A comparison between *J. nigra* and DB indicated distinct taxa, including *GOUTA6 and Gematimonas* in *J. nigra* and *Granulicella* and *Subgroup_2_ge* (Supplementary Figure 4b). Additionally, DESeq analysis was validated using ANCOM (Supplementary Figure 5a).

A total of 275 fungal community indicator OTUs were detected across our three categories: *J. nigra* (133 OTUs), *Q. rubra* (65 OTUs), and DB (65 OTUs) (Supplementary Table 3). The presence of *J. nigra* correlated with the *Hymenogastraceae* and *Ceratobasidiaceae* families, and the *Coniochaeta* genus, whereas *Q. rubra* showed elevated *Basidiomycota*, predominantly comprising ectomycorrhizal (EcM) fungi. Conversely, *J. nigra* led to a decrease in the percentage of *Ascomycota*, while *Q. rubra* exhibited reductions in *Glomeromycota* and *Chytridiomycota*. DESeq analysis demonstrated significant differences in fungal taxa within the *J. nigra*-*Q. rubra* comparison (Figure 5b), which was further validated using ANCOM (Supplementary Figure 5b). Specifically, *Q. rubra* enriched EcM fungal taxa such as *Tuber*, *Inocybe*, *Tomentella*, *Amanita*, and *Russula*, while *J. nigra* favored *Hymenogaster*, *Lobulomyces*, *Thanatephorus*, *Otospora*, and *Entrophospora*. These variations extended to comparisons with DB (Supplementary Figures 3c,d).

### 3.5 Tree species affect microbial co-occurrence network properties and taxa hubs

**Figure 6:**
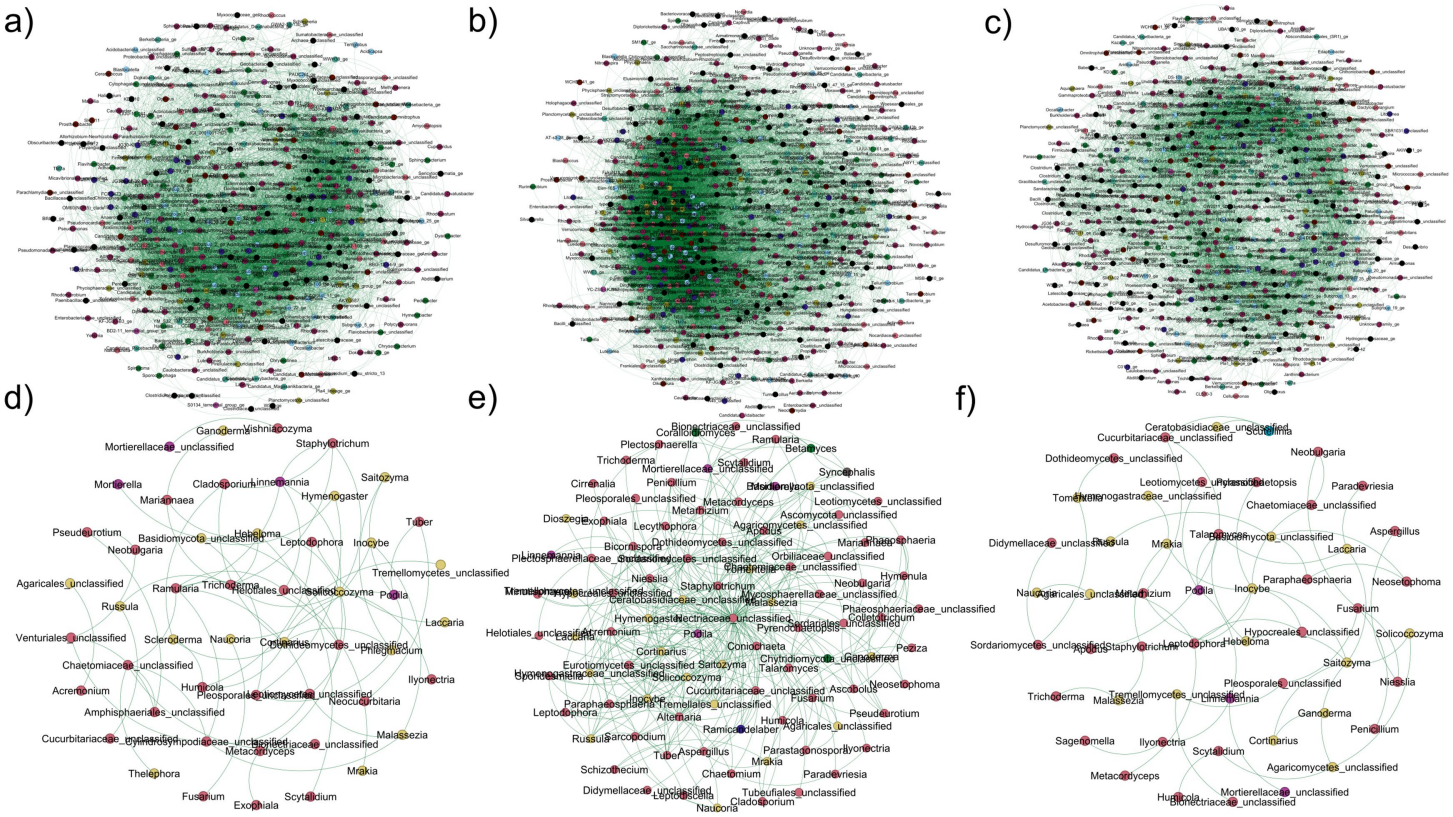
Co-occurrence patterns in bacterial/archaeal **(a–c)** and fungal **(d–f)** microbiomes in **(a,d)** *J. nigra*, **(b,d)** *Q. rubra*, and **(c,e)** DB. Connections represent strong (Spearman’s *ρ* > 0.6) and significant (FDR-corrected *p* < 0.01) correlations. Node size corresponds to the OTUs’ degree and edge thickness corresponds to Spearman’s correlation coefficient value. Node size of individual nodes corresponds to the OTUs’ degree, and the thickness of connections between noted (edges) corresponds to Spearman’s correlation coefficient value. DB, non-plant soil; BW, *J. nigra*; RO, *Q. rubra*.

The soil bacterial/archaeal community network exhibited distinct co-occurrence patterns for each tree species and the DB category (Figure 6; Table 2). BD presented a higher number of nodes and edges than both tree species. Additionally, BD presented values for density, clustering coefficient, and avg. number of neighbors (Table 2). *Q. rubra* presented the highest mean degree and average path length values (Table 2). Among the identified keystones for bacterial networks under different plant species, 42 taxa belonging to five phyla were identified, including *Sphingomonas*, *Arenimonas*, *Bryobacter*, *Bradyrhizobium*, *Ellin6067*, and *Subgroup_2_ge* (Supplementary Table 4).

**Table 2:** Topological features of the co-occurrence networks constructed based on bacteria and fungi.

Kingdom	Tree species	Edges	Nodes	Clustering coefficient	Avg. path length	Density	Mean degree	Avg. number neighbors
Bacteria/archaea	*J. nigra*	9,218	464	0.51	2.44	0.085	0.315	39.56
	*Q. rubra*	7,324	551	0.41	2.64	0.048	0.382	26.57
	DB	16,252	551	0.55	2.39	0.094	0.245	54.93
Fungi	*J. nigra*	72	54	0.21	4.28	0.023	0.560	2.00
	*Q. rubra*	58	55	0.15	5.03	0.028	0.746	1.63
	DB	375	74	0.24	1.98	0.083	0.275	7.81

Similarly, both tree species experienced reduced nodes and edges in the soil fungal community network, resulting in a smaller mean degree and clustering coefficient (Figure 6d; Table 2). Notably, the *Q. rubra* network exhibited the highest clustering coefficient and modularity (Figure 6d; Table 2). The *Nectriaceae* and *Ceratobasidiaceae* families were the only ones identified as a keystone taxon in the DB network. Additionally, *Saitozyma* presented high values of mean degree and betweenness centrality in both *Q. rubra* and DB specifically. Finally, *Tuber* was presented in both tree plant species (Supplementary Table 4). Furthermore, other keystone taxa identified in *Q. rubra* were *Inocybe*, *Talaromyces*, *Laccaria*, and *Podila* while in *J. nigra* appeared *Solicoccozyma*, *Staphylotrichum*, *Cladosporium*, *Phlegmacium*, and *Cortinarius* (Supplementary Table 4).

### 3.6 Effects of tree species, season, and soil depth on environmental parameters and associated microbial communities

Results from a Mantel test revealed the significant influence of soil variables on microbial community structures. Specifically, soil pH (*r* = 0.3, *p* = 0.001) significantly affected bacterial/archaeal community structure. Additionally, pH (*r* = 0.3, *p* = 0.001), TN (*r* = 0.18, *p* = 0.003), and TC (*r* = 0.077, *p* = 0.044) collectively shaped fungal community structure. Humidity was significantly correlated with *Ascomycota* (*r* = 0.38, *p* = 0.006) and *Kickxellomycota* (*r* = −0.36, *p* = 0.011) among fungal phyla. In contrast, *Glomeromycota* and *Kickxellomycota* exhibited negative correlations with pH, TOC, TC, and TN (Supplementary Table 5). The C/N ratio also demonstrated a significant correlation with *Murocomycota* (*r* = −0.33, *p* = 0.021).

Chemical properties represented by TOC, TC, and TN positively correlated with Proteobacteria, Bacteroidota, and *TM7* to a significant degree. These same chemical properties were negatively correlated with *Acidobacteriota*, *Firmicutes* (only TOC), *Planctomycetota*, and *Chloroflexi* (Supplementary Table 5). A significant correlation was observed between pH and several phyla, including *Proteobacteria*, *Acidobacteriota*, *Actinobacteriota*, *Firmicutes*, and *Armatimonadota*. The C/N ratio impacted *Bacteroidota*, *Actinobacteriota*, and *Chloroflexi* while soil humidity showed correlations with *Bacteroidota* and *Chloroflexi* (Supplementary Table 5).

## 4 Discussion

Tree species composition can drastically reshape soil microbial communities and consequently induce suppression or acceleration of soil functions (Chen et al., 2019; Ramirez et al., 2019). Therefore, understanding how different tree species influence soil microbial communities is imperative for elucidating these dynamics. In our work, we revealed significant distinctions in soil microbial communities associated with *J. nigra*, *Q. rubra*, and the DB. Specifically, building upon previous research by Gardner et al. (2023), our results underscore how the introduction of trees, particularly *J. nigra* and *Q. rubra*, significantly modified and enriched microbial diversity within the DB soil structure. Notably, an impressive 47 and 56% of bacterial/archaeal and fungal OTUs appeared only in the plots with trees. These results support our principal hypothesis that tree species were the strongest influencers of microbial communities. Furthermore, the distribution of bacterial/archaeal and fungal OTUs revealed that *J. nigra* harbored a higher proportion of these taxa compared to other species. Tedersoo et al. (2013) showed that *J. nigra* in agroforestry systems enhanced soil bacterial/archaeal diversity, particularly with increasing tree biomass and agroforestry age in a previous study. Also, litter from *J. nigra* can improve vital soil fertility indicators, such as C and N content, promoting abundance and diversity principally within bacterial/archaeal communities (Hong-ye et al., 2016). In contrast, fungal communities associated with both *J. nigra* and *Q. rubra* displayed a higher species diversity compared with soil without trees. The presence of *J. nigra* led to the highest increase in diversity within bacterial/archaeal communities.

Our findings revealed distinct responses of bacterial and fungal communities to different tree species, consistent with prior studies (Lu-Irving et al., 2019; Onufrak et al., 2020). This observation highlights that tree species exert varying degrees of influence on bacterial and fungal communities, shedding light on the complex interactions between plants and soil microbes. However, these results contrast previous studies, where soil depth was the main driver of bacterial community (Prada-Salcedo et al., 2022). In our study, depth and season had a significant but limited impact on bacterial and fungal communities. The influence of soil depth on microbial communities is a result of the nutrient gradient effect. Specifically, soil depth influenced *Acidobacteriota*, with higher levels detected in the deeper soil profiles (Eilers et al., 2012) where nutrient availability is often less abundant (DeAngelis et al., 2009). The seasonal effects mainly affect two important variables for soil microbial communities: humidity and temperature. Our samplings were carried out in autumn and spring when the climate is not extreme like in summer or winter, thus the effect on the microbial communities might be less influential or need more days to show significant changes detectable by technique (Gardner et al., 2023).

Bacterial community analysis showed *Proteobacteria* and *Acidobacteriota*, common in forest soil, were the most abundant phyla recovered in this study, a finding that aligns with observations by other researchers (Uroz et al., 2016). Additionally, this analysis detected a notable abundance of *Bacteroidota*, *Verrucomicrobiota*, and *Actinobacteriota*. *J. nigra* significantly increased the abundance of *Bacteroidota* and *Proteobacteria* in comparison to *Q. rubra*. In contrast, Onufrak et al. (2020) showed that the *Spirosomaceae* family (*Bacteroidota*) appeared as an indicator of *J. nigra.* Both phyla are well-known for their copiotrophic life strategy, different from oligotrophs in that they thrive in highly organic, nutrient-rich soils (Fierer et al., 2007; Prada-Salcedo et al., 2022). Conversely, *Q. rubra* increased the representation of *Acidobacteriota* members, a phylum characterized by their survival ability in low C availability or oligotrophic environments (Fierer et al., 2007). The shift is supported by the positive correlation between C and N stocks with *Proteobacteria* and the negative correlation with *Acidobacteriota*, supporting the theory of life strategy attributes. The decline in pH in *Q. rubra* soils allows for *Acidobacteriota* populations to rise.

Bacterial taxa in forests and plantations have been documented extensively (Uroz et al., 2016; Landesman et al., 2014). The substantially higher proportion of *Subgroup_2_ge*, *Granulicella*, *Bryobacter*, *Occallatibacter*, and *Candidatus_Solibacter* can be explained by the positive relationship between *Acidobacteriota* and the decreased soil pH found in *Q. rubra* (Fierer et al., 2007). Within the *Q. rubra* plots, we also observed a significant abundance of genera such as *Burkholderia*, *Sphingomonas*, and *Rhizomicrobium*, which can be associated with the robust growth of these trees (Landesman et al., 2014; Morrison et al., 2014). These genera play crucial roles in N cycling as nitrogen-fixing bacteria (Anderson and Habiger, 2012) and in phosphorous solubilization (Halder et al., 1990). Additionally, Kuramae et al. (2019) indicated a significant positive correlation between *Acidobacteriota* and nitrogen-fixing bacteria (*Rhizobiales* and *Burkholderiales*) and decomposers (*Elsterales* and *Xanthomonadales*). The enhancement of two *Bacteroidota* genera, *Flavobacterium* and *Chryseobacterium*, known for their occurrence in root associations and deadwood, was linked to *J. nigra*. This is consistent with the association of *Bacteroidota* with carbon-rich soils like those found around *J. nigra* (Fierer et al., 2012) and their diverse physiological capabilities, including ligninolytic activity (Dilly et al., 2001; Kolton et al., 2013).

Soil-associated fungal communities vary with tree species (Baldrian, 2017). In this study, the fungal community primarily featured phyla such as *Basidiomycota*, *Ascomycota*, and *Mortierellomycota*. Our findings align with prior studies indicating that *Ascomycota* and *Basidiomycota* dominate forest and plantation soils (Urbanova et al., 2015). Specifically, *J. nigra* increased the abundance of *Glomeromycota*, while *Q. rubra* favored *Basidiomycota* and *Ascomycota*. Several studies suggest that the uptick in Basidiomycota is linked to their capacity for decomposing more recalcitrant litter (Nguyen et al., 2016). In contrast, *Glomeromycota* represents AM fungi which can be associated with *J. nigra* (Štursová et al., 2016).

The identity of plant species remains a principal factor shaping AM fungal communities (Mummey and Rillig, 2006; Baldrian, 2017). Our findings suggest host preference in symbiotic EcM fungal species, with *Q. rubra* hosting the following genera: *Inocybe*, *Amanita*, *Tomentella*, *Lactarius*, and *Russula*, whereas *J. nigra* was more commonly associated with *Hebeloma*, *Trichoderma*, *Coniochaeta*, *Coralloidiomyces*, and *Enterocarpus*, as reported in previous research (Zhang and Wang, 2017). Previous research has emphasized the impact of N supplies and soil pH on EcM community diversification (Suz et al., 2014). Additionally, some studies have shown the crucial role of EcM fungi in accessing organic nutrients, particularly under acidic conditions (Averill et al., 2019), which is congruent with our results here. Moreover, the increased abundance of EcM fungi in *Q. rubra* is consistent with the greater EcM richness observed in these plots compared to *J. nigra* and DB. Furthermore, when comparing *J. nigra* with DB, *J. nigra* exhibited a greater abundance of various EcM fungi, including *Hebeloma*, *Laccaria*, *Hymenogasters*, and *Protoglossum*. In contrast to saprophytic fungal communities, tree host specialization is a significant force influencing EcM fungal populations (Ferlian et al., 2018; Chen et al., 2019).

Microorganisms thrive in complex associations rather than in isolation (Banerjee et al., 2019). To explore this, we created distinct co-occurrence networks for bacterial/archaeal and fungal communities and analyzed their features to understand how plant species affect microbial co-occurrence patterns. Our findings revealed that the DB zone presented the highest number of connections (edges) between the different nodes for bacterial/archaeal and fungal co-occurrence networks. Additionally, DB networks presented the highest values for clustering coefficient and density parameters. On the contrary, the *Q. rubra* presented higher values for the network average path length and mean degree parameters. This observation suggests that greater microbial diversity does not necessarily lead to more complex co-occurrence networks. This phenomenon can be explained by the ecological theory of the species-energy relationship (Evans et al., 2005; Gaston, 2000; Wright, 1983). *J. nigra*’s richer C and N environment provides a diverse range of energy sources for bacterial/archaeal communities, enhancing their diversity. Consequently, bacterial/archaeal species are more dependent on environmental energy and nutrients than on complex interactions with other bacterial species (Tu et al., 2020).

Keystone taxa are highly linked microorganisms that play essential roles in the structure and function of the microbiota and operate as environmental indicators (Banerjee et al., 2019). In our analysis, we identified keystone taxa belonging to *Proteobacteria* associated with tree species’ plantations, *Arenimonas*, *Sphingomona*s, *KF-JG30-C25_ge*, and *Oxalobacteraceae_unclassified* for *J. nigra*. These findings highlight the specific bacterial taxa that may be crucial in maintaining microbial community stability under varying soil conditions. This response pattern is consistent with the soil pH decrease produced by these trees. Additional keystone taxa associated with trees were *Actinobacteriota* which have a significant impact on the structure and function of forest ecosystems (Lupatini et al., 2014) due to their role in the degradation of cellulitic materials and the solubilization of P (Mitra et al., 2022; Zhou et al., 2020). Furthermore, *Sphingomonas* is capable of degrading very complex carbon substrates including both substituted and unsubstituted mono- and poly-aromatic hydrocarbons up to five rings (Jones et al., 2011), giving this genus a distinctive advantage in decomposing recalcitrant carbon sources such as lignin. When we analyzed fungal keystone taxa, *Tuber*, belonging to the *Pezizales* order, was a common keystone taxon in both plant species and DB. This fungus is integral to the forest food web, forming EcM associations that are most widely distributed in forests of the northern hemisphere (Obase et al., 2011; Bahram et al., 2013). Conversely, *Mortierella* emerged as a keystone taxon associated with *J. nigra*, a genus known for its saprotrophic capabilities (Yadav et al., 2015). These findings emphasize the divergent ecological roles that fungal communities may play depending on the host tree species.

Our findings show that tree species significantly influence C and N stocks and pH levels (Gardner et al., 2023). Variations in these soil properties can be attributed to differences in tree physiology and their subsequent impact on soil chemistry. Existing research strongly supports the notion that forest trees contribute to soil acidification over decadal intervals (Binkley, 1995; Mitchell et al., 2010). In addition, our study unveiled significant correlations between soil properties and microbial community structures, echoing findings from previous research (Lauber et al., 2009; Banerjee et al., 2019). These correlations underscore the intricate interplay between environmental factors and microbial community dynamics in soil ecosystems. Specifically, the correlation between total C and N with the fungal community illustrates the essential role of fungi in the C and N cycle. Fungi are the primary agents involved in the decomposition of soil organic matter in temperate forests (Baldrian, 2008). However, it is important to note that bacterial and fungal communities are not governed by the same environmental parameters (Uroz et al., 2016). Notably, fungal communities are strongly influenced by tree species, while bacterial communities are primarily influenced by root exudates (Urbanova et al., 2015).

One of our work’s limitations is that *J. nigra* and *Q. rubra* associate with different types of mycorrhizas, which introduces variability in fungal community analysis. The fungal primers used here are not suitable for sequencing AM fungi. As a result, our findings may include a greater number of mycorrhizal fungi that are more closely related to *Q. rubra* than *J. nigra*. Future studies should incorporate primers that effectively capture the entire spectrum of soil mycorrhizal communities associated with these tree species to achieve a more comprehensive analysis. Although observational study designs limit causal inference, they are beneficial for exploring an understudied system and generating suggestions to guide future microbiome research on hardwood trees. Another potential limitation of our work is the use of OTUs instead of amplicon sequence variants (ASVs). Previous studies suggest that the diversity index based on ASV may outperform those based on OTUs (Joos et al., 2020). Although rarefaction and clustering level-identity OTUs appear to bridge the gap between OTU- and ASV-based techniques (Chiarello et al., 2022), we rarified the samples in this study to be consistent. Future research should consider the adoption of ASV techniques to improve the accuracy of microbial diversity assessments.

## 5 Conclusion

In summary, this study provides compelling evidence of the strong impact of tree species on soil properties and soil bacterial/archaeal and fungal communities. Our findings demonstrate that tree species differ significantly in shaping belowground communities. Specifically, tree species, characterized by distinct litter quality and mycorrhiza fungal associations, impact not only the taxonomy of bacterial/archaeal and fungal communities but also their diversity and interactions through interspecific connections. Moreover, tree species directly or indirectly alter soil properties (Sommer et al., 2017). These changes in soil properties can either enhance or diminish a forest’s resilience to climate-related stressors such as drought, diseases, and pests (Buma and Wessman, 2013; Williams and Ginzel, 2020). Consequently, those tree species able to adapt to the modified soil conditions may thrive, while others may struggle, thereby causing significant impacts on the overall resilience and diversity of the forest ecosystem. Looking ahead, research is necessary to associate the soil microbial communities with plant health and development. Such studies are crucial to determine which microbial species can enhance early tree plantation growth thereby ensuring greater plant growth and yields.

## Data Availability

The datasets presented in this study can be found in online repositories. The names of the repository/repositories and accession number(s) can be found in the article/Supplementary material.

## Supplementary material

SUPPLEMENTARY FIGURE 1 Rarefaction curves of observed species plotted against sampling depth for **(a)** bacteria/archaea and **(b)** fungi.

SUPPLEMENTARY FIGURE 2 Venn diagrams illustrating the shared OTUs of the **(a)** fungal and **(b)** bacterial/archaeal microbiomes associated with *J. nigra*, *Q. rubra*, and DB. The diagrams show the absolute number of OTUs shared between microbiomes for both tree species and DB across seasons and depths. DB, non-plant soil; BW, *J. nigra*; RO, *Q. rubra*.

SUPPLEMENTARY FIGURE 3 Relative abundance of fungal functional guilds in soils associated with both tree species and the DB environment. DB, non-plant soil; BW, *J. nigra*; RO, Northern red oak.

SUPPLEMENTARY FIGURE 4 Differential representation of significant abundance OTUs between *J. nigra* and *Q. rubra* at the genus level for **(a,b)** bacterial/archaeal and **(c,d)** fungal taxa. Figures **(a)** and **(c)** compare *J. nigra* vs. DB, while **(b)** and **(d)** compare *Q. rubra* vs. DB. Dots represent taxa, colors indicate phylum, and labels represent genus. Multiple points per genus represent distinct species of a given genus. Positive values indicate a positive response to *J. nigra* or *Q. rubra*, while negative values indicate a positive response to DB. Only OTUs with differential abundance at *p* < 0.05 are represented. DB, non-plant soil; BW, *J. nigra*; RO, Northern red oak.

SUPPLEMENTARY FIGURE 5 Differential representation of significant abundance OTUs, based on ANCOM, between *J. nigra* and *Q. rubra* at the genus level for **(a)** bacterial/archaeal and **(b)** fungal taxa. Positive values indicate a positive response to *Q. rubra*. Negative values indicate a positive response to *J. nigra.* Only the probabilities with a differential abundance at *p* < 0.05 are represented.
