# Supplementary figures and images for: Effects of tree species identity on soil microbial communities in Juglans nigra and Quercus rubra plantations
Source: Front Microbiol. 2024 Oct 29;15:1442026. doi: 10.3389/fmicb.2024.1442026 (PMC11554539; doi:10.3389/fmicb.2024.1442026)

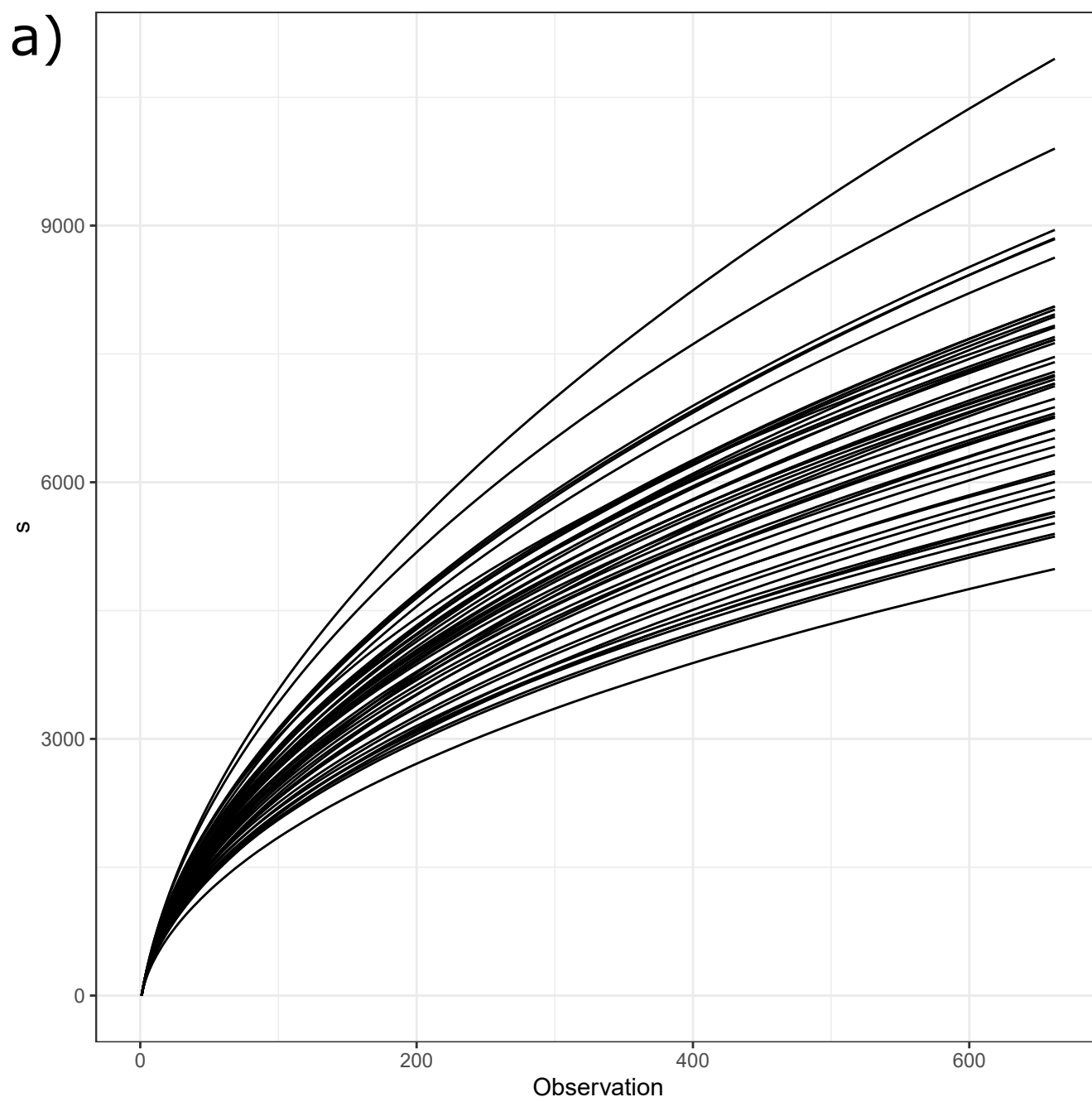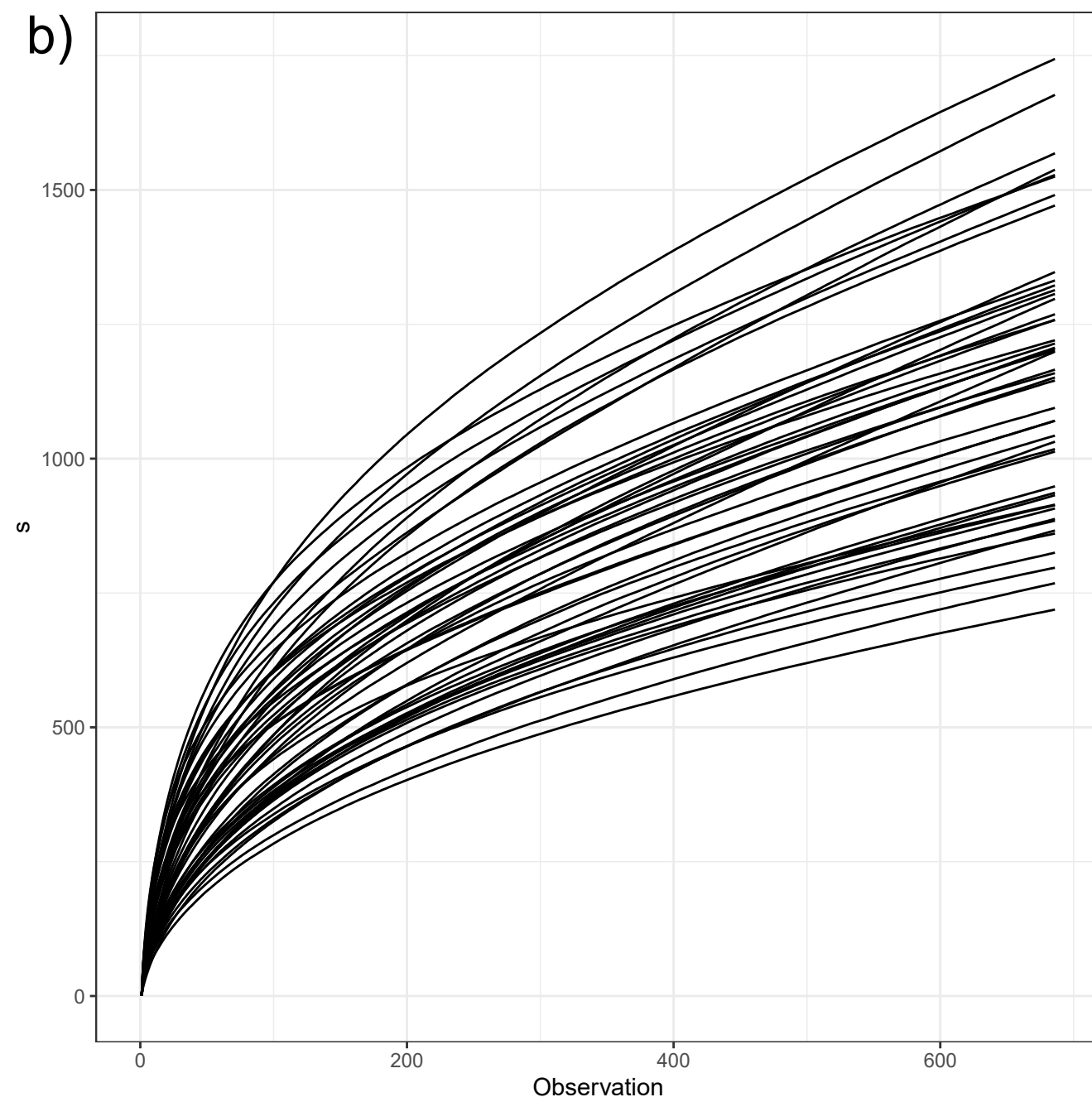

Supplement: SUPPLEMENTARY FIGURE 1 — Rarefaction curves of observed species plotted against sampling depth for (a) bacteria/archaea and (b) fungi. [file Image_1.PDF]

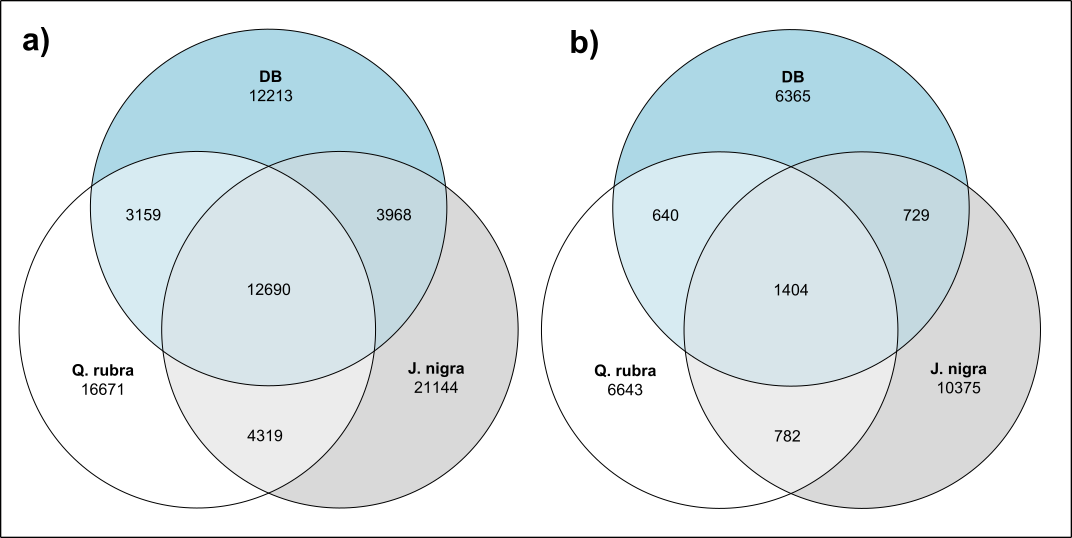

Supplement: SUPPLEMENTARY FIGURE 2 — Venn diagrams illustrating the shared OTUs of the (a) fungal and (b) bacterial/archaeal microbiomes associated with J. nigra, Q. rubra, and DB. The diagrams show the absolute number of OTUs shared between microbiomes for both tree species and DB across seasons and depths. DB, non-plant soil; BW, J. nigra; RO, Q. rubra. [file Image_2.TIFF]

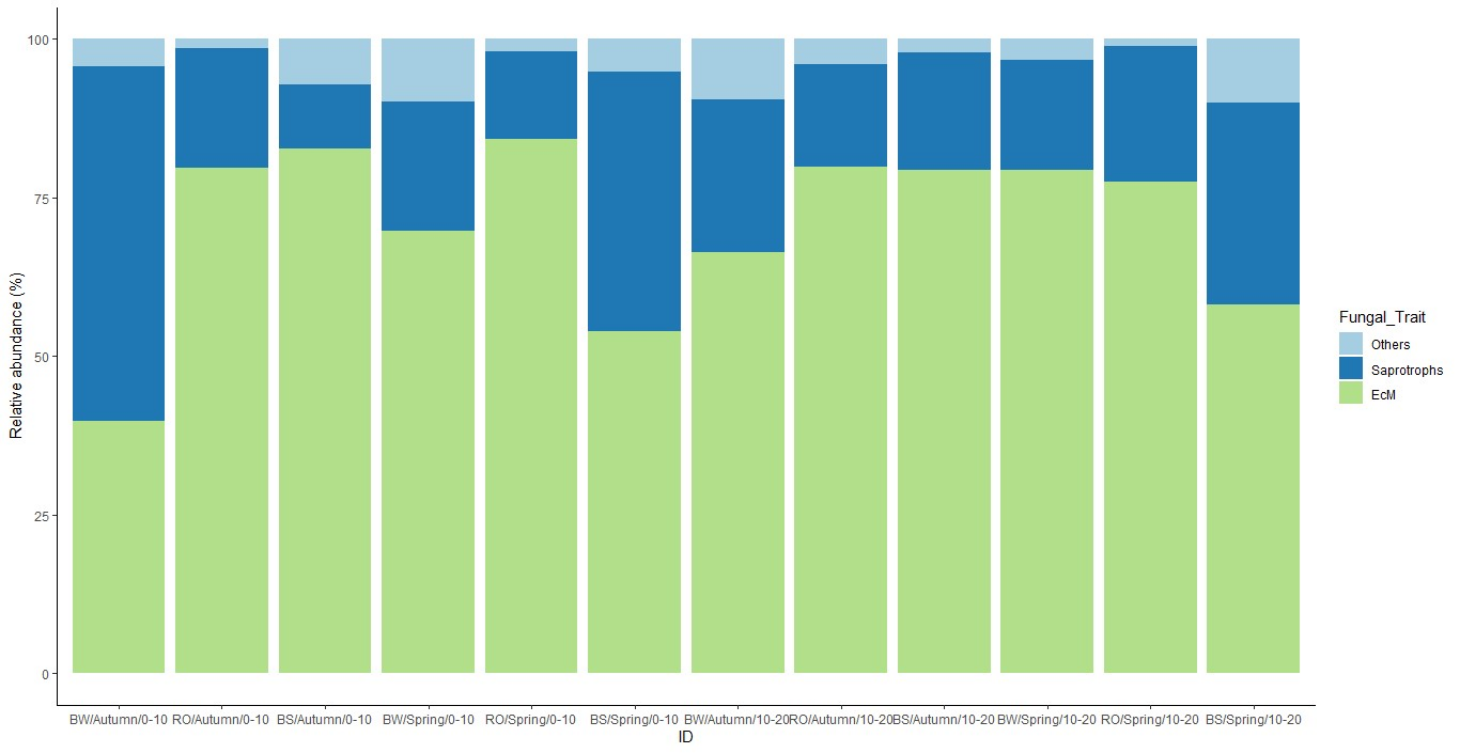

Supplement: SUPPLEMENTARY FIGURE 3 — Relative abundance of fungal functional guilds in soils associated with both tree species and the DB environment. DB, non-plant soil; BW, J. nigra; RO, Northern red oak. [file Image_3.TIFF]

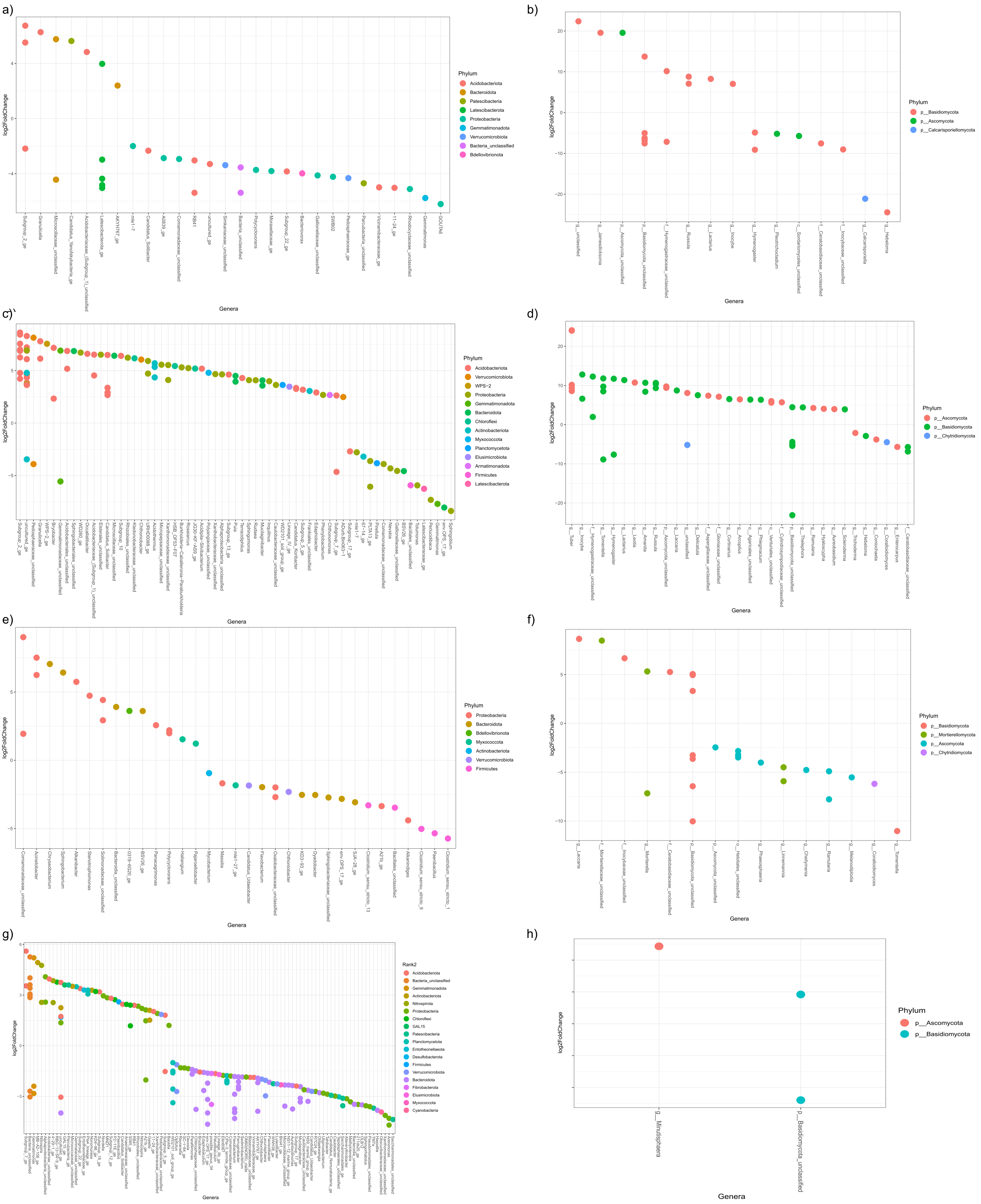

Supplement: SUPPLEMENTARY FIGURE 4 — Differential representation of significant abundance OTUs between J. nigra and Q. rubra at the genus level for (a,b) bacterial/archaeal and (c,d) fungal taxa. Figures (a) and (c) compare J. nigra vs. DB, while (b) and (d) compare Q. rubra vs. DB. Dots represent taxa, colors indicate phylum, and labels represent genus. Multiple points per genus represent distinct species of a given genus. Positive values indicate a positive response to J. nigra or Q. rubra, while negative values indicate a positive response to DB. Only OTUs with differential abundance at p < 0.05 are represented. DB, non-plant soil; BW, J. nigra; RO, Northern red oak. [file Image_4.TIFF]

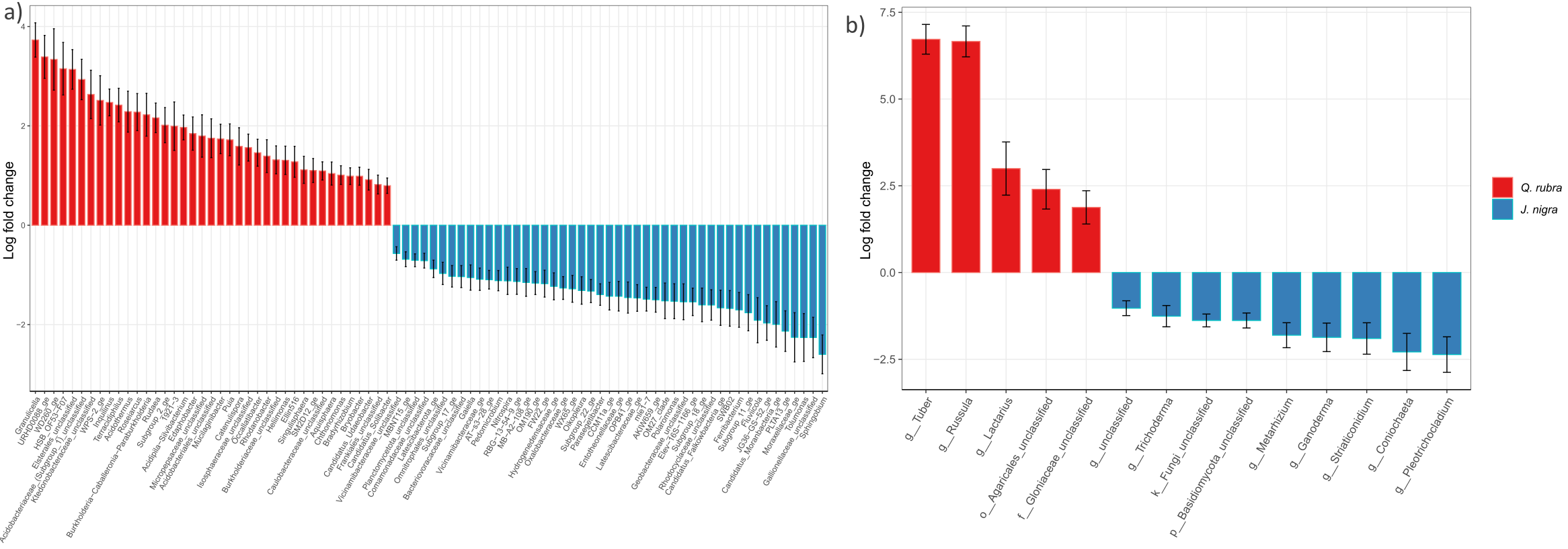

Supplement: SUPPLEMENTARY FIGURE 5 — Differential representation of significant abundance OTUs, based on ANCOM, between J. nigra and Q. rubra at the genus level for (a) bacterial/archaeal and (b) fungal taxa. Positive values indicate a positive response to Q. rubra. Negative values indicate a positive response to J. nigra. Only the probabilities with a differential abundance at p < 0.05 are represented. [file Image_5.TIFF]
